# Expression of angiopoietin-like protein 2 in ovarian tissue of rat polycystic ovarian syndrome model and its correlation study

**DOI:** 10.1186/s12958-020-00651-7

**Published:** 2020-09-25

**Authors:** Dandan Wang, Yihong Guo, Shujuan Chai, Ke Shen, Yanchun Li, Ruiqin Zhao

**Affiliations:** 1 Reproduction Center, The Third Affiliated Hospital of Henan University of Traditional Chinese Medicine, Zhengzhou, Henan China; 2 Reproductive and Genetic Hospital, The First Affiliated Hospital of Zhengzhou University, Zhengzhou, Henan China

**Keywords:** Polycystic ovary syndrome, PI3K/Akt, Angiopoietin-like protein 2, Metformin

## Abstract

**Background:**

This study investigated the expression of angiopoietin-like protein 2 (ANGPTL2) in the tissues of rat models of polycystic ovary syndrome (PCOS) and its correlation with PCOS.

**Methods:**

Six-weeks-old female specific pathogen-free rats (*n* = 60) were divided into blank control, PCOS model, and metformin groups (*n* = 20/group). After 21 days of metformin intervention, the serum sex hormones, fasting blood glucose, fasting insulin, and insulin resistance (IR) of rats in each group were measured. The mRNA levels of ANGPTL2, Foxol, and Akt in the ovarian tissues were monitored by real-time fluorescence quantitative PCR.

**Results:**

Compared with the control group, the levels of serum sex hormones, fasting blood glucose, fasting insulin, and IR in the model group showed significant increases, and the levels of ANGPTL2, Foxol, and Akt in the ovarian tissue also showed significant increases. Compared with the PCOS group, the serum sex hormones, fasting blood glucose, fasting insulin, and IR of rats in the metformin group were significantly decreased, and the levels of ANGPTL2, Foxol, and Akt in ovarian tissues also showed significant decreases.

**Conclusions:**

These findings suggest that ANGPTL2 might participate in the development of PCOS through the PI3K/Akt signaling pathway. Metformin improves IR by reducing the expression of ANGPTL2, thus improving the endocrine environment of PCOS and might change the disease outcome.

## Background

Polycystic ovary syndrome (PCOS) is one of the most common endocrine, metabolic disorders in women of childbearing age, and it is regarded as a frequent cause of ovulation disorders and infertility in women [1], as well as cardiovascular disease, diabetes, and endometrial cancer [2]. The incidence of PCOS has been reported to be 4–10% [3]. At present, there is no unified opinion about the etiology of PCOS. Insulin resistance (IR) plays an important role in the development of PCOS [4]. Glintborg et al. [5] showed that women with PCOS have ovarian dysfunction, indicating systemic and local IR in the ovarian tissue of PCOS patients.

PCOS ovarian IR mainly occurs due to the insulin signal transduction pathway, which in turn is closely related to the impairment of key molecules of insulin, L-phosphatidylinositol 3-kinase (PI3K), and glucose transporter 4 signaling pathway [6, 7]. PI3K/serine-threonine protein kinase (Akt) is an insulin-related signaling pathway, and insulin activates the downstream PI3K for regulating glucose metabolism and activates the downstream signaling molecule Akt [8]. The activated Akt regulates the biological activity of the cells [9]. Decreased phosphorylation of the PI3K/Akt signaling pathway is considered to be an important feature of IR in patients with PCOS [10]. The levels of insulin in ovulation-stimulating follicular fluid of patients with PCOS is significantly higher than that of normal women, and the levels of insulin in patients with PCOS and IR is higher than that of non-IR patients, indicating IR in the ovaries in patients with PCOS and abnormal PI3K/Akt signaling [11]. Fox is a downstream protein of Akt kinase, which binds to DNA, and enhances or inhibits transcription [12]. FoxO1 protein is a subgroup of fox factors and is highly expressed in the granulosa cells of mammalian follicles. FoxO1 plays an important role in the proliferation of ovarian granulosa cells, and so its expression level plays an important role in follicular development and oocyte maturation [13].

The development of follicles not only requires the regulation of hormones and signal transduction but also requires several blood vessels to provide adequate nutrition. Angiopoietin-like proteins (ANGPTLs) are closely related to angiogenesis [14–16]. Currently, there are very few studies on the role of ANGPTLs in follicular development. In adipose tissues, ANGPTL2 mediates chronic inflammation of adipose tissue and promotes obesity-related IR [17], but whether ANGPTL2 mediates IR through the PI3K/Akt signal transduction pathway in affecting the follicular development has not been reported previously.

Hence, in this study, the mRNA differential expression of ANGPTL2, PI3K, Akt, and FoxO1 was detected in ovarian tissues between rat models of PCOS treated or not with metformin by real-time fluorescence quantitative polymerase chain reaction (qRT-PCR), investigated whether ANGPTL2 affects the development of follicles through the PI3K/Akt signaling pathway in the PCOS models, and investigated the correlations between ANGPTL2 and PCOS and IR.

## Methods

### Animals and grouping

A total of 60 female Sprague-Dawley (SD) specific pathogen-free rats aged 3-weeks-old with a body mass of 50 ± 20 g were obtained from the animal experiment center of Zhengzhou University (production license No. SCXK (Yu) 2017–0001). The rats were kept in an experimental animal center of the Third Affiliated Hospital of Henan University of Traditional Chinese Medicine. All rats were housed in special cages, and standard feed and special water for rats were provided ad libitum. The padding in the cages was changed twice a week. The temperature of the experimental animal center was maintained at 22 ± 1 °C, and the humidity was about 30%. A light/dark cycle was provided alternatively for 12 h/day. The SD rats (*n* = 60) were adaptively fed for 1 week. The rats were then divided into the normal control group (NC) with 20 rats and the experimental group with 40 rats using a random number table. The rats in the NC group were fed with standard chow and provided water ad libitum. This experiment was approved by the ethics committee of the Henan University of Traditional Chinese Medicine, and a statement on informed consent from the client or owner.

The rats in the experimental group were given letrozole (1 mg/kg.d) dissolved in 1% carboxymethyl fiber by gavage for 21 days. The smear of vaginal exfoliated cells was observed every day from week 1 after the model was established. During the period of gavage, the feed and water were provided ad libitum for rats. Their body weight was measured every 2 days. From day 7 after gavage, the rats were examined for vaginal secretion and stained with methylene blue every morning, and the estrous cycle was determined by analyzing the cytological changes. The following four items should be achieved for the successful establishment of the PCOS rat model: 1) body weight change; 2) changes in serum hormone levels, especially changes in testosterone (T) and luteinizing hormone (LH); 3) disappearance of the estrus cycle according to the vaginal smears; and 4) polycystic changes in ovarian tissue. After 24 h of model establishment, normal saline at 0.01 ml/g/d was administered to the rats of the PCOS group, while a metformin solution at 0.01 ml/g/d was given to the rats in the metformin group for 21 days continuously.

### Specimen collection and storage

The rats were kept fasting from 8:00 p.m. and weighed at 8:00 a.m. on the next morning. Chloral hydrate (10%, 0.3 ml/100 g) was intraperitoneally injected for anesthesia. Blood (5–8 ml) was collected from the abdominal aorta and centrifuged at 3000 r/min for 10 min after holding for 30 min. The serum was stored at − 80 °C. The bilateral ovaries of the rats were separated; one ovary was maintained in liquid nitrogen for 2 h and frozen at − 80 °C, while the other ovary was fixed in 4% paraformaldehyde.

### qRT-PCT

The mRNA expression of Angptl2, Akt, and Foxol was detected by PCR. The mRNA was extracted by trituration, and the concentration and purity were determined. According to the reverse transcription kit instructions, the reverse transcription system was prepared for a total volume of 20 μL. The reverse transcription conditions were as follows: 50 °C for 2 min, 95 °C for 10 min, 95 °C for 30 s, and 60 °C for 30 s, for 40 cycles. The primers, specific to rats, were manufactured by Wuhan Qing Ke Biotechnology Co., Ltd. (Wuhan, China) and are listed in Table 1.

**Table 1:** Sequences of the PCR primers

Name	Primer	Sequence	Size
Rat GAPDH	Forward	5′- ATGGGTGTGAACCACGAGA − 3′	229 bp
	Reverse	5′- CAGGGATGATGTTCTGGGCA − 3′	
Rat Akt	Forward	5′- CTGCCCTTCTACAACCAGGA − 3′	214 bp
	Reverse	5′- CATACACATCCTGCCACACG − 3′	
Rat Foxo1	Forward	5′- AAGAGCGTGCCCTACTTCAA − 3′	188 bp
	Reverse	5′- GCTCTTCTCCGGGGTGATTT − 3′	
Rat Angptl2	Forward	5′- ACAACCGCATCATCAACCAG −3’	250 bp
	Reverse	5′- GGGTCATGTCTCTGGTCACA −3’	

### Histology

The fixed ovaries were sectioned at 4 μm and routinely stained with hematoxylin and eosin (Beyotime Institute of Biotechnology, Haimen, China), according to the manufacturer’s instructions. The sections were photographed at 100× using a light microscope (Nikon, Tokyo, Japan).

### Western blotting

The expression levels of Angptl2, Akt, and Foxol in ovarian tissues were detected by western blot. The total proteins were extracted with the RIPA buffer (Beyotime Institute of Biotechnology, Haimen, China), the protein concentration was determined using the BCA assay, and 40 μg/lane of proteins were separated by SDS-PAGE and transferred to PVDF membranes. Primary antibodies (1:1500) against p-AKT (ab38449, Abcam, Cambridge, UK), p-Foxo1 (ab131339, Abcam), and ANGPTL2 (ab36014, Abcam) were incubated overnight at 4 °C, followed by 1 h with the HRP-conjugated anti-rabbit secondary antibody (1:2000). An ECL kit (Pierce Chemicals, Dallas, TX, USA) was used, and the images were analyzed using the ImageJ software.

### Statistical analysis

SPSS 22.0 statistical software was used for data analysis, and the measurement data were represented as means ± standard deviations. One-way analysis of variance (ANOVA) was performed for multiple comparisons of the mean. The least significant difference (LSD) method was performed for post hoc multiple comparisons in case of homogeneity of variance, while the Dunnett T3 method was performed for post hoc multiple comparisons in case of unequal variances. *P* < 0.05 was considered to be statistically significant.

## Results

### Comparison of serum sex hormones among groups

The serum LH, follicle-stimulating hormone (FSH), and T of rats in the PCOS and metformin groups were higher (*P* < 0.05) compared with the normal control group. The serum levels of LH, FSH, and T in the metformin group were lower than those of the PCOS group (*P* < 0.05). No significant difference was observed in the LH/FSH ratio among the three groups (*P* > 0.05) (Table 2).

**Table 2:** Comparison of serum sex hormones among the groups

Group	n	LH (IU/L)	FSH (IU/L)	T (IU/L)	LH/FSH
Normal control group	20	5.45 ± 0.40	3.00 ± 0.30	0.42 ± 0.31	1.79 ± 0.22
PCOS group	20	7.59 ± 0.20^a^	4.70 ± 0.40^a^	1.28 ± 0.32^a^	1.62 ± 0.25
Metformin treatment group	20	6.73 ± 0.46^b^	4.97 ± 1.05^b^	1.04 ± 0.21^b^	1.46 ± 0.37

Compared with the normal control group, ^a^*P* < 0.05; compared with the PCOS group, ^b^*P* < 0.05

**Fig. 1:**
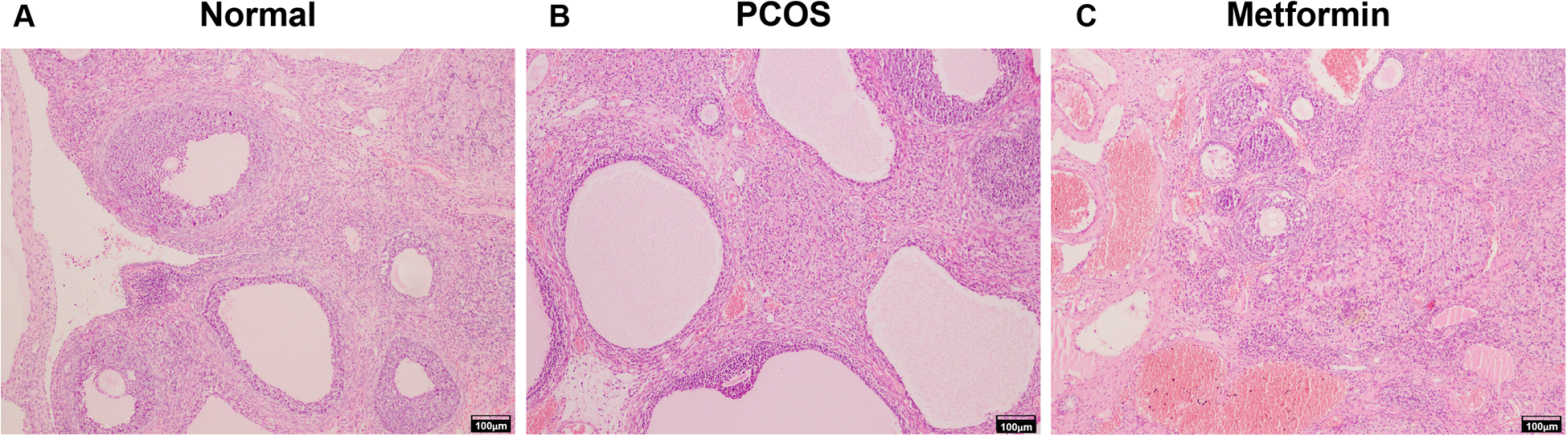
Hematoxylin and eosin staining of ovarian tissues from control normal rats, rat models of polycystic ovarian syndrome (PCOS), and rat models of PCOS treated with metformin. Scale bar = 100 μm

HE staining of ovarian tissue sections under a low-power microscope (Fig. 1) showed that rats with PCOS had a disorderly ovarian tissue structure, and there were multiple follicles with cystic dilatation under the capsule. Oocytes were not found in some follicles, luteum was rare, and the granulosa cell layers were reduced, with usually 2–3 layers. The follicles in the ovaries of rats in the normal control group were at different stages of development, with multiple corpus luteum, close and orderly arrangement of granulosa cells, complete morphology, and up to 8–9 layers. The ovarian tissue of the metformin group also showed follicles at different stages of development, with part of the corpus luteum and tight arrangement of granulosa cells.

### Fasting blood glucose, fasting insulin, and IR among the groups

Compared with the normal control group, the fasting blood glucose, fasting insulin, and HOMA-IR levels in the PCOS and metformin groups were significantly higher (*P* < 0.05). The fasting blood glucose, fasting insulin, and HOMA-IR in the metformin group were lower than those in the PCOS group (Table 3).

**Table 3:** Fasting blood glucose, fasting insulin and insulin resistance (IR) of rats among the groups

Group	n	Fasting blood glucose (mmol/l)	Fasting insulin (mIU/l)	HOMA-IR
Normal control group	20	7.26 ± 1.02	28.36 ± 5.04	9.34 ± 1.03
PCOS group	20	11.15 ± 0.72^a^	47.28 ± 15.42^a^	23.26 ± 6.83^a^
Metformin treatment group	20	7.93 ± 0.89^b^	32.06 ± 7.64^b^	13.82 ± 3.71^b^

Compared with the normal control group, ^a^*P* < 0.05; compared with the PCOS group, ^b^*P* < 0.05

### Expression levels of ANGPTL2, p-Akt, and p-Foxol mRNA in ovarian tissue among the groups

**Fig. 2:**
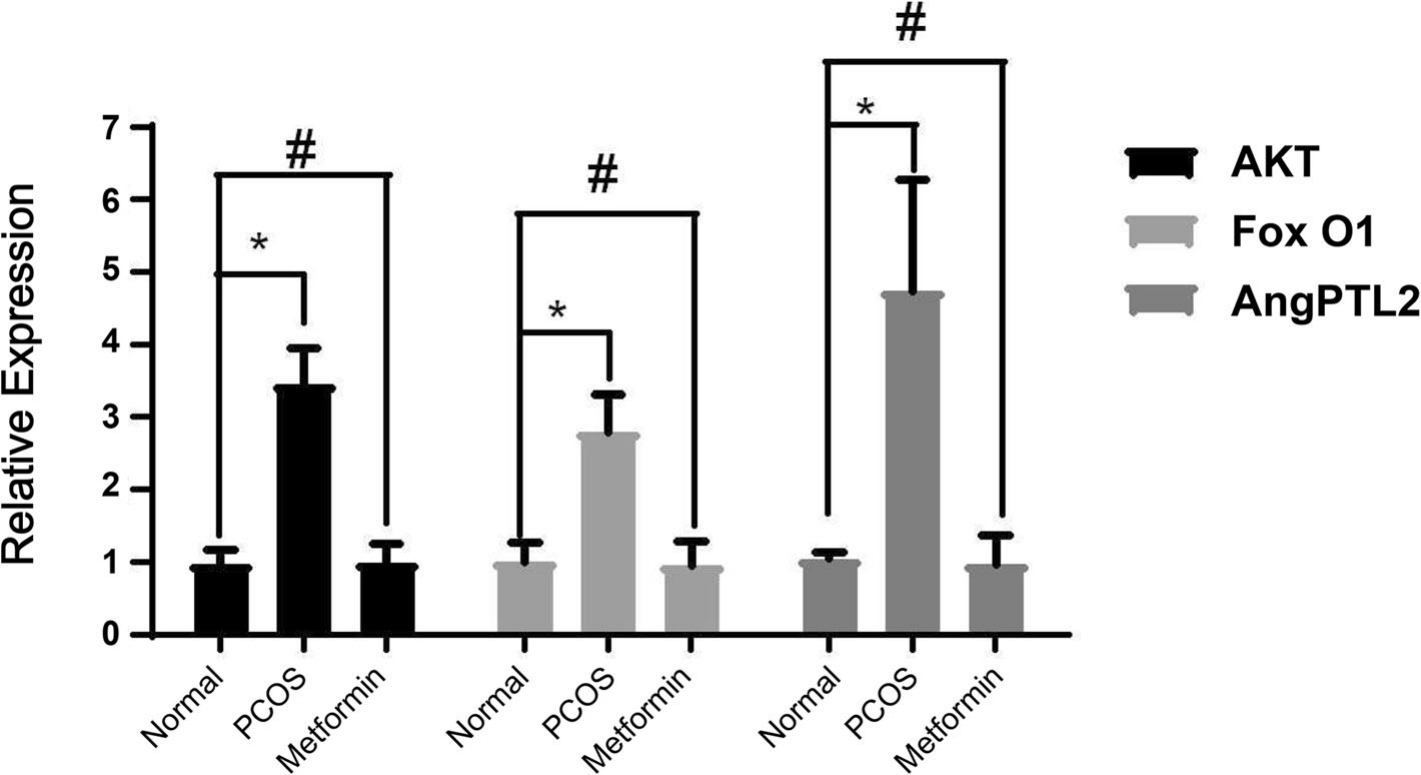
Relative expression of FoxoI, Akt, and ANGPTL2 in the ovaries of control normal rats, rat models of polycystic ovarian syndrome (PCOS), and rat models of PCOS treated with metformin. *P < 0.05; ^#^*P* < 0.05 vs. the normal control group

**Fig. 3:**
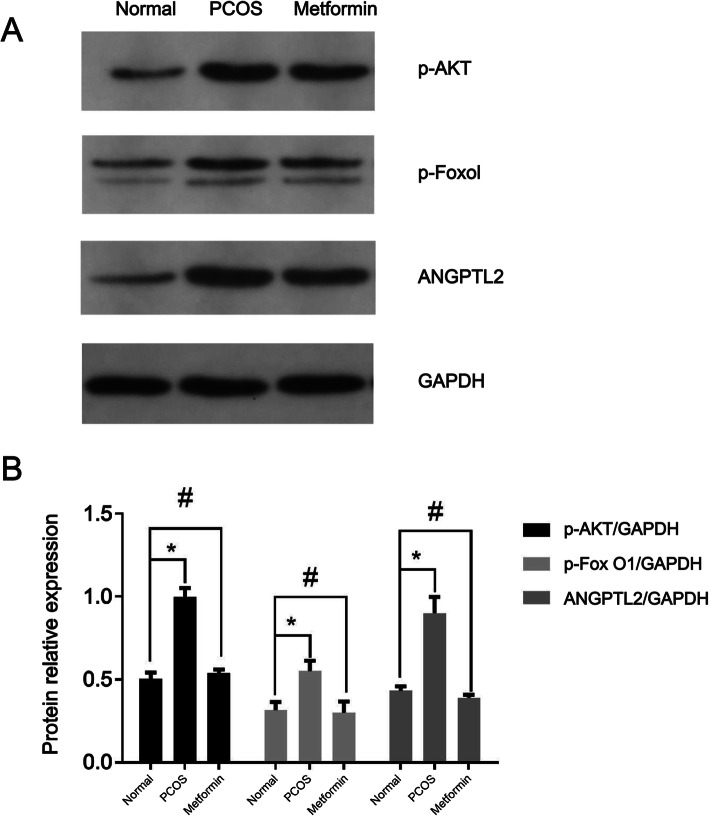
Western blots (**a**) and quantification analysis (**b**) of p-Akt, p-Foxo1, and ANGPTL2 in the ovaries of control normal rats, rat models of polycystic ovarian syndrome (PCOS), and rat models of PCOS treated with metformin. *P < 0.05; ^#^*P* < 0.05 vs. the normal control group

The expression levels of ANGPTL2, p-Akt, and p-Foxol in the PCOS group were significantly higher than those in the control group (*P* < 0.05). Compared with the PCOS group, the expression levels of ANGPTL2, p-Akt, and p-Foxol in the ovarian tissue of the metformin group were significantly lower (*P* < 0.05) (Fig. 2). Compared with the blank group, the protein expression levels of ANGPTL2, p-Akt, and p-Foxol in the ovarian tissues of the PCOS group were higher than that of the control group (*P* < 0.05). Compared with the PCOS group, the expression levels of ANGPTL2, p-Akt, and p-Foxol in the ovarian tissues of the metformin group were significantly reduced (*P* < 0.05) (Fig. 3a-b).

## Discussion

Follicular development is influenced by a series of sex hormones from the hypothalamus - pituitary - ovarian axis control [18], but also by the local ovarian tissue through autocrine and paracrine actions in the form of a series of related factors involved in the process follicular development [19], and thus the changes in the ovarian microenvironment might promote or inhibit on follicular development. The process of follicular development and maturation involves neovascularization, and the process of angiogenesis depends on the participation of the family members of ANGPTLs. ANGPT1 in the ANGPTLs family can affect the production of steroid hormones, reduce premature ovarian failure, and promote the proliferation of mouse sinusiform follicles. It is speculated that ANGPT1 may regulate the ovarian function by regulating the stability of ovarian blood vessels [20]. ANGPTL1 and ANGPTL2 are highly homologous to ANGPT1 and ANGPT2 in structure and function, so it is speculated that ANGPTLs may have a certain protective effect on the vasculature system of the ovary, but the specific mechanism is not clear. Doi et al. [21] suggested that increased serum levels of the ANGPTL2 protein in the general population showed a positive correlation with the pathogenesis of type 2 diabetes. Xue et al. [22] detected the differential expression of the ANGPTL1 and ANGPTL2 genes in the cumulus-oocyte complexes from metaphase II (CC_MII_) of patients with PCOS, and the results showed that only ANGTPTL2 was highly expressed, indicating that ANGPTL2 more likely affects the development and maturation of cumulus granules in patients with PCOS. This was the main reason as to why this study focused on ANGPTL2.

In this study, the serum sex hormone, fasting blood glucose, fasting insulin, and IR were significantly increased in the PCOS and metformin groups, and the histological examination of the ovaries revealed abnormalities characteristic of PCOS, indicating the successful establishment of the PCOS rat model. RT-PCR showed that the expression levels of ANGPTL2 were low in the control group, while high in the PCOS group. After metformin treatment, the levels of ANGPTL2 were decreased, strongly suggesting the participation of ANGPTL2 in the occurrence of PCOS. It was also found that the phosphorylation level of these proteins was increased in the PCOS group. Therefore, ANGPTL2 might play a role in follicular development through the PI3K/Akt signaling pathway.

Previous studies showed that FOXO1 could upregulate the expression of pro-apoptotic proteins such as Bin and P27^kip1^, induce follicular atresia, promote the apoptosis of granulosa cells, and inhibit PI3K/Akt signaling pathway activation, increasing the expression of FOXO1 and promoting the generation of apoptosis signals [23]. This study found that the expression of local ANGPTL2 in the ovarian tissues of rats in the PCOS group was abnormally increased, which might subsequently aggravate IR, promote PI3K phosphorylation, increase FOXO1phosphorylation, down-regulate apoptotic gene expression, and induce follicular atresia, causing PCOS.

ANGPTL2 was reported as a key inflammatory mediator derived from the adipose tissue, and the changes in ANGPTL2 protein levels in blood circulation could be used as a marker for metabolic abnormalities to induce obesity [24]. It is worth mentioning that the elimination of ANGPTL2 improves the inflammatory response and IR of adipose tissue in diet-induced obese mice, while the overexpression of the ANGPTL2 gene in the adipose tissue of non-obese mice leads to local inflammatory response and systemic IR [24].

Based on the above theory, metformin treatment decreased the expression of ANGPTL2 mRNA when compared with the PCOS model group (*P* < 0.05), indicating that metformin might improve IR by reducing the expression of ANGPTL2. This, in turn, improves the endocrine environment of PCOS and promotes follicle development. Of course, this mechanism will have to be examined more closely before this hypothesis can be confirmed. The present study explored the possibility that ANGPTL2 might be associated with PCOS, and the results showed that ANGPTL2 is involved. Nevertheless, the exact mechanisms and the other proteins involved still need to be determined. Future studies should look into the protein expression of ANGPTL2.

## Conclusions

ANGPTL2 plays an important role in the occurrence and development of PCOS through the PI3K/Akt signaling pathway, aggravating IR, and increasing the expression of apoptotic genes. Nevertheless, the specific molecular mechanisms have to be further studied.

## Data Availability

The datasets used and/or analyzed during the current study are available from the corresponding author on reasonable request.

## Abbreviations

PCOS: Polycystic ovary syndrome
IR: Insulin resistance
ANGPTLs: Angiopoietin-like proteins
CCD: Coiled-coiled district
FLD: Fibrinogen-like district
ARP: Angioprotein-related proteins
SD: Sprague-Dawley
NC: Normal control group
LSD: Least significant difference
FSH: Follicle-stimulating hormone
